# Derivatives of 3-Aminopyrazine-2-carboxamides: Synthesis, Antimicrobial Evaluation, and in Vitro Cytotoxicity

**DOI:** 10.3390/molecules24071212

**Published:** 2019-03-28

**Authors:** Ghada Bouz, Lucia Semelková, Ondřej Janďourek, Klára Konečná, Pavla Paterová, Lucie Navrátilová, Vladimír Kubíček, Jiří Kuneš, Martin Doležal, Jan Zitko

**Affiliations:** 1 Faculty of Pharmacy in Hradec Králové, Charles University, Heyrovského 1203, 500 05 Hradec Králové, Czech Republic; lucia.semelkova@seznam.cz (L.S.); jando6aa@faf.cuni.cz (O.J.); konecna@faf.cuni.cz (K.K.); navratl2@faf.cuni.cz (L.N.); kubicek@faf.cuni.cz (V.K.); kunes@faf.cuni.cz (J.K.); dolezalm@faf.cuni.cz (M.D.); 2 Department of Clinical Microbiology, Faculty Hospital, Sokolská 581, 500 05 Hradec Králové, Czech Republic; pavla.paterova@fnhk.cz

**Keywords:** aminopyrazine, antibacterial activity, antifungal activity, antimycobacterial activity, cytotoxicity, pyrazinamide derivatives

## Abstract

We report the design, synthesis, and in vitro antimicrobial activity of a series of *N*-substituted 3-aminopyrazine-2-carboxamides with free amino groups in position 3 on the pyrazine ring. Based on various substituents on the carboxamidic moiety, the series is subdivided into benzyl, alkyl, and phenyl derivatives. The three-dimensional structures of the title compounds were predicted using energy minimization and low mode molecular dynamics under AMBER10:EHT forcefield. Compounds were evaluated for antimycobacterial, antibacterial, and antifungal activities in vitro. The most active compound against *Mycobacterium tuberculosis* H37Rv *(Mtb)* was 3-amino-*N*-(2,4-dimethoxyphenyl)pyrazine-2-carboxamide (**17**, MIC = 12.5 µg/mL, 46 µM). Antimycobacterial activity against *Mtb* and *M. kansasii* along with antibacterial activity increased among the alkyl derivatives with increasing the length of carbon side chain. Antibacterial activity was observed for phenyl and alkyl derivatives, but not for benzyl derivatives. Antifungal activity was observed in all structural subtypes, mainly against *Trichophyton interdigitale* and *Candida albicans*. The four most active compounds (compounds **10**, **16**, **17**, **20**) were evaluated for their *in vitro* cytotoxicity in HepG2 cancer cell line; only compound **20** was found to exert some level of cytotoxicity. Compounds belonging to the current series were compared to previously published, structurally related compounds in terms of antimicrobial activity to draw structure activity relationships conclusions.

## 1. Introduction

Tuberculosis (TB) is a serious infection known for its lethality for centuries. Despite the fact that the incidence of TB has been slowly decreasing since the beginning of the millennium, there are around 10 million new TB cases every year [1]. Since 2016, TB is the most common cause of death among all infectious diseases, followed by HIV [1]. The World Health Organization (WHO) has set a target to reduce the incidence of TB by 90% and the number of TB death cases by 95% by the year 2035 [2]. TB-HIV co-infection and the emergence of multidrug-resistant mycobacterial strains constitute a growing risk that challenges the management of TB [3,4]. Generally, antimicrobial resistance (AMR) is recognized as a major threat to sustainable development goals defined by the United Nations [1]. The need for the development of new antimicrobial compounds effective against drug-resistant pathogens is generally recognized.

**Figure 1:**
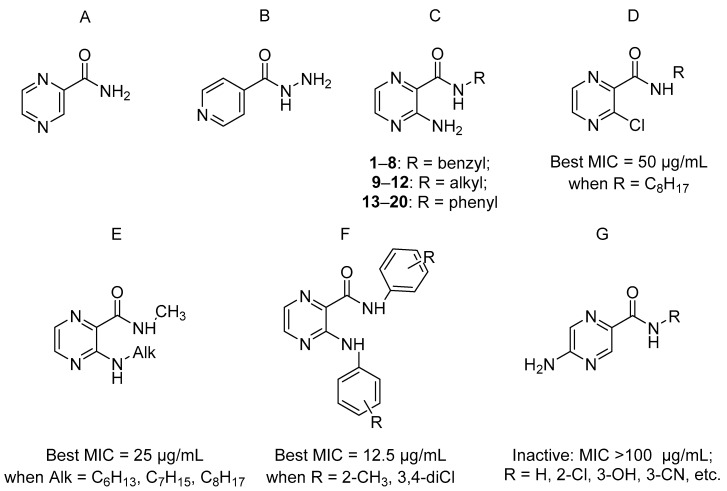
General structures of the discussed compounds (A: pyrazinamide; B: isoniazid (INH); C: current series; D: [14]; E: [14]; F: [15]; G: [16]) and best MICs against *Mtb* H37Rv.

Pyrazinamide (PZA, Figure 1) is a first line anti-tubercular agent. Due to its sterilizing effect on semi-dormant tubercle bacilli, PZA itself contributes to shortening the duration of TB treatment from 9–12 months to 6 months [5]. PZA is considered to be a prodrug that is converted to its active form, pyrazine-2-carboxylic acid (pyrazinoic acid, POA), by mycobacterial pyrazinamidase/nicotinamidase. The intracellular accumulation of POA was considered to cause the acidification of mycobacterial cytoplasm and the subsequent collapse of membrane potential and transport [3,5]. This structurally non-specific mechanism of action (MoA) was recently disputed by experimental data showing that such effects of PZA/POA are negligible at concentrations lower than 10x the minimum inhibitory concentration (MIC) [2]. It was revealed that POA and/or PZA itself affects the biosynthesis of mycobacterial mycolic acids via inhibition of fatty acid synthase (FAS) I [6,7], interferes with the biosynthetic pathway of acetylcoenzyme A by inhibiting aspartate decarboxylase (PanD) [8], and disrupts the nicotinamide pathway by inhibition of quinolinic acid phosphoribosyl transferase (QAPRTase) [9]. POA was suggested to inhibit *trans*-translation by binding to ribosomal protein S1 (RpsA) [10,11]. However, this mechanism of action (MoA) was also recently disputed [12]. This clearly demonstrates the ongoing interest in PZA and its MoA. New information regarding potential subcellular targets of PZA can be exploited for rational design of PZA derivatives with improved pharmacokinetic/pharmacodynamic properties. In addition, several amides and esters of POA have been prepared as prodrugs as an attempt to overcome mycobacterial resistance since their activation is dependent on enzymes other than the typical pyrazinamidases [13].

As a part of our long-term project focused on structural modifications of PZA, we present the current series of twenty 3-aminopyrazine-2-carboxamides with various substituents on the carboxamidic nitrogen. This series is the last block in the investigation of the effect of different substituents at position 3 on the pyrazine ring (free amino group vs. chlorine atom [14] vs. *N*-alkyl/*N*-benzyl substituted amino group [14,15]) on the antimicrobial activity (Figure 1). The current *N*-substituted 3-aminopyrazine-2-carboxamide series is further subdivided based on the R substituent into eight benzyl derivatives (compounds **1**–**8**), four alkyl derivatives (compounds **9**–**12**), and eight phenyl derivatives (compounds **13**–**20**) (Figure 1). We have previously synthesized a number of aminopyrazinamide derivatives with free amino groups at position 5 on the pyrazine ring, yet they possessed no significant antimycobacterial activity [16].

All prepared compounds were evaluated in vitro for their antimycobacterial activities against five different mycobacterial strains. As a complementary test, the compounds were screened for antibacterial and antifungal activity against strains of clinical importance. Active compounds were evaluated for in vitro cytotoxicity in the HepG2 liver cancer cell line. We theoretically predicted the 3D structures of prepared compounds and evaluated their potential to form intramolecular hydrogen bonds. Aminopyrazines exert a wide range of biological activities; particularly, compound **4** was evaluated as an allergy inhibitor [17], and compound **13** was found to exert inhibitory effects on Ataxia Telangiectasia and Rad3 Related (ATR) protein kinase [18]. 

## 2. Results and Discussion

### 2.1. Synthesis

**Scheme 1:**
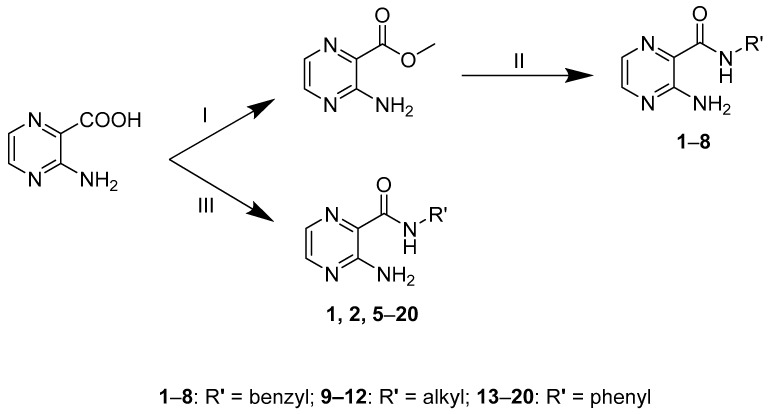
Synthesis of *N*-substituted 3-aminopyrazine-2-amides. Procedure A: I) H_2_SO_4_, methanol, 48 h, rt; II) substituted benzylamine, NH_4_Cl, methanol, MW: 130 °C, 40 min, 90 W [19]; Procedure B: III) 1. CDI, DMSO; 2. benzylamine/alkylamine/aniline, MW: 120 °C, 30 min, 100 W [20]. For R’ refer to Table 2.

Two different procedures were used for converting the starting 3-aminopyrazine-2-carboxylic acid into the corresponding carboxamides (Scheme 1). In procedure A, the first step was a Fisher esterification in the presence of H_2_SO_4_ and methanol to afford the corresponding methyl ester. The second step was the aminolysis of the obtained ester by corresponding benzylamine using microwave irradiation [19]. In procedure B, the starting acid was treated with the coupling agent 1,1′-carbonyldiimidazole (CDI) in anhydrous dimethyl sulfoxide (DMSO) [20]. CDI reacts with the acid to form the activated intermediate (3-aminopyrazin-2-yl)(1*H*-imidazol-1-yl)methanone that facilitates the following formation of the desired amide. The reaction of the activated acid with corresponding amines was performed using microwave irradiation. Both procedures A and B are two step reactions. In procedure A, the intermediate methyl ester was prepared in bulk, isolated, stored, and used for several subsequent reactions to produce the final *N*-substituted carboxamides. On contrary, in procedure B the intermediate of activated acid was not isolated and was immediately used in subsequent reactions. Benzyl derivatives **1**–**2** and **5**–**8** were prepared by both procedures to compare the efficiency of amide formation from the ester (method A) and amide formation directly from the acid activated by CDI (method B). Table 1 summarizes the obtained yields by both procedures. Except for compound **5**, procedure B consistently gave higher yields, and thus was determined to be the optimum procedure to obtain the intended amides. Compounds **9**–**20** were then prepared by procedure B.

The final compounds were isolated mainly as pale yellow to yellow solids. Final compounds were fully characterized by their ^1^H- and ^13^C-NMR spectra, IR spectra, melting point and elemental analysis. The data were fully consistent with the proposed structures. In the IR spectra, the final compounds showed a signal at 1641–1684 cm^−1^, attributed to the amidic carbonyl. In the ^1^H-NMR spectra, the signal of the amidic hydrogen was dependent on the solvent used; it appeared at 8.80–10.85 ppm in DMSO-*d*_6_ and at 7.88–9.81 ppm in CDCl_3_. The two signals of the pyrazine hydrogens H5 and H6 appeared at 7.76–8.30 ppm, independent of the solvent. The signal of the two 3-amino hydrogens appeared as a broad singlet at 7.49–7.62 ppm in DMSO-*d*_6_, while for compounds measured in CDCl_3_, the signal was undetectable in some compounds. In the ^13^C-NMR spectra, the signal of the amidic carbon appeared at 163.35–166.64 ppm. Representative NMR spectra (of compounds **5**, **9**, and **14**) are attached as Supplementary Material.

### 2.2. Predicted 3D Structure

Energetically minimized 3D structures (conformations) of the title compounds were predicted by MOE v2018.1001 (Molecular Operating Environment, Chemical Computing Group, Montreal, QC, Canada). All calculations were performed under AMBER10:EHT forcefield (implicit R-field solvent model). Compounds were imported as SMILES and initially minimized using combined steepest gradient and conjugate gradient method to 0.1 kcal mol^−1^A^−2^. These initially minimized conformations were subjected to conformational search using low mode molecular dynamics with default settings to determine the global minimum for each compound. The resulting conformer with lowest energy for each compound was considered a representative structure.

**Figure 2:**
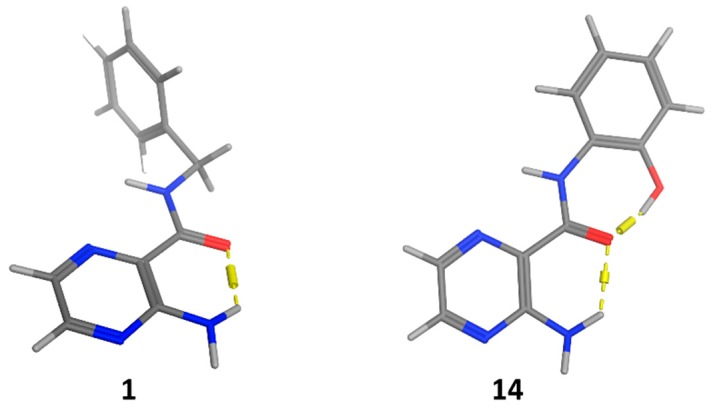
Lowest energy conformers of compounds **1** and **14** with visualization of intramolecular hydrogen bonds.

As we expected, all conformers with the lowest energy contained *trans* amide bonds. Conformers with *cis* amide bonds possessed much higher energy, usually 6–7 kcal/mol higher than the corresponding *trans* carboxamides. In accordance with our previous study on similar substituted derivatives of 3-aminopyrazine-2-carboxamides [15], we observed an intramolecular hydrogen bond (IMHB) formed between the amino group (donor) and the carboxamidic oxygen (acceptor), see Figure 2. This *H*-bond was present in lowest energy conformations of all title compounds of this paper – all benzyl, phenyl and alkyl derivatives. Additionally, compound **14** contained another intramolecular *H*-bond between hydroxyl (donor) in position 2 of the benzene ring and the carboxamidic oxygen (acceptor), see Figure 2.

IMHBs of this type have several implications towards the physico-chemical properties and potential pharmacodynamic properties (binding to receptor) of the compounds. Hydrogens and lone electron pairs involved in IMHBs are not readily available for forming *H*-bonds with molecules of water as a solvent, therefore such compounds are less polar and less water soluble (more lipophilic) than they appear. To calculate their log *P*, one should use a method capable of (at least partial) evaluation of these intramolecular bonding effects. The simplest methods for log *P* prediction based only on fragmentation methods do not give accurate results [21]. In ChemDraw, the Clog*P* algorithm (in contrast to the log*P* algorithm) takes into account some intramolecular interactions as we demonstrated in our previous publication [15]. IMHBs also limit molecules’ ability to bind to their potential receptors – hydrogens and lone electron pairs forming IMHBs are not directly available for forming *H*-bonds with receptors. IMHBs can be broken to enhance the molecule’s potential to interact with solvent and/or the receptor, but this process is connected with energy penalty, which needs to be considered to correctly evaluate whether the process of exchange of intramolecular interaction for intermolecular interaction is favorable [22,23]. The presence of IMHBs also effects the conformational variability of the title compounds–conformers with the described intramolecular *H*-bond are preferred and energetically favorable. Taken together, the possibility to form intramolecular *H*-bond affects significantly both physico-chemical properties of title compounds and the molecular recognition process between them and their potential biological receptor(s).

As an attempt to verify the presence of the *in silico* predicted IMHB, we assumed that in an ^1^H-NMR experiment the presence of IMHB would affect the shift of the hydrogen involved in the IMHB (hydrogen from the amino group in position 3). This effect would be dependent on the temperature, as with increased temperature the stability of the IMHB would be decreased and both of the amino hydrogens would become non-bonded and therefore spectroscopically equivalent. The instrumentation used was the same as described in Section 3.1. Firstly, we measured the ^1^H-NMR spectra of compound **1** in DMSO-*d*_6_ at temperatures 25–115 °C (in 10 °C increments). However, we did not observe the expected phenomenon of merging the signals of the two amino hydrogens at elevated temperature. This might be explained by the used solvent (DMSO-*d*_6_), which can form H-bonds to the solvated compound and interfere with the formation of the IMHB. Therefore, as a second attempt, we repeated the measurement in deuterated PhMe (solvent not capable of forming H-bonds) and we have successfully observed the predicted phenomenon. At 25 °C, the signals for the amino hydrogens were 8.24 ppm (bs, 1H, hydrogen atom supposedly involved in the IMHB) and 4.82 ppm (bs, 1H, hydrogen atom not involved in the IMHB). With increasing temperature, the signals moved to each other and at 80 °C they were merged in one signal at 6.41 ppm (bs, 2H, NH_2_). The signal of the amidic hydrogen (CONH) remained stable throughout the used range of temperatures (7.90 ppm at 25 °C, 7.86 ppm at 80 °C). We consider these results a preliminary confirmation of the presence of the discussed IMHB.

### 2.3. Antimycobacterial Activity

The prepared compounds were tested for *in vitro* whole cell growth inhibitory activity against five mycobacterial strains, namely *Mycobacterium tuberculosis* H37Rv (*Mtb*), *M. avium*, *M. kansasii*, *M. smegmatis* and *M. aurum*. *M. smegmatis* and *M. aurum* are, unlike the others, rapid growing strains that do not cause overt tuberculosis infection in humans [24]. Yet since these two strains are safer to handle and have genotype and phenotype sufficiently similar to *Mtb*, they are used as surrogate models [25]. Antimycobacterial activity is expressed as minimal inhibitory concentration (MIC) in µg/mL and summarized in Table 2. 

Four benzyl derivatives (compounds **3**, **4**, **7**, **8**) exerted weak antimycobacterial activity against *Mtb* (MIC = 50–100 µg/mL). These compounds have monosubstitution on the benzene ring in position 4 (compounds **3**, **4**, **8**), or position 3 (compound **7**). The benzyl derivatives with two substituents on the benzene ring (**5**: R’ = 2,4-diOCH_3_ and **6**: R’ = 3,4-diCl) were inactive (MIC > 100 µg/mL). When comparing the disubstituted benzyl derivatives to their corresponding phenyl derivatives of the same substituents, it was found that the phenyl derivatives were active unlike the benzyl derivatives (**5** vs. **17**; **6** vs. **19**). The most active compound against *Mtb* was the phenyl derivative **17** with R’ = 2,4-diOCH_3_. 

Based on our experience from the previously published series of alkylamino substituted pyrazinamide derivatives, the antimycobacterial activity was highest for compounds possessing side chains with six to eight carbons [26,27,28,29]. In the alkyl derivatives presented in this paper (compounds **9**–**12**), octylamide (compound **12**: MIC = 25 µg/mL, 100 µM) exerted the highest activity, followed by heptylamide (**11**: MIC = 50 µg/mL, 212 µM) and hexylamide (**10**: MIC = 100 µg/mL, 450 µM). Similarly, the antimycobacterial activity against *M. kansasii* increased with increasing length of the carbon chain (**10**: MIC = 100 µg/mL, 450 µM; **11**: MIC = 50 µg/mL, 212 µM; **12**: MIC = 25 µg/mL, 100 µM). No other compounds presented in the manuscript had significant activity against *M. kansasii*. 

**Figure 3:**
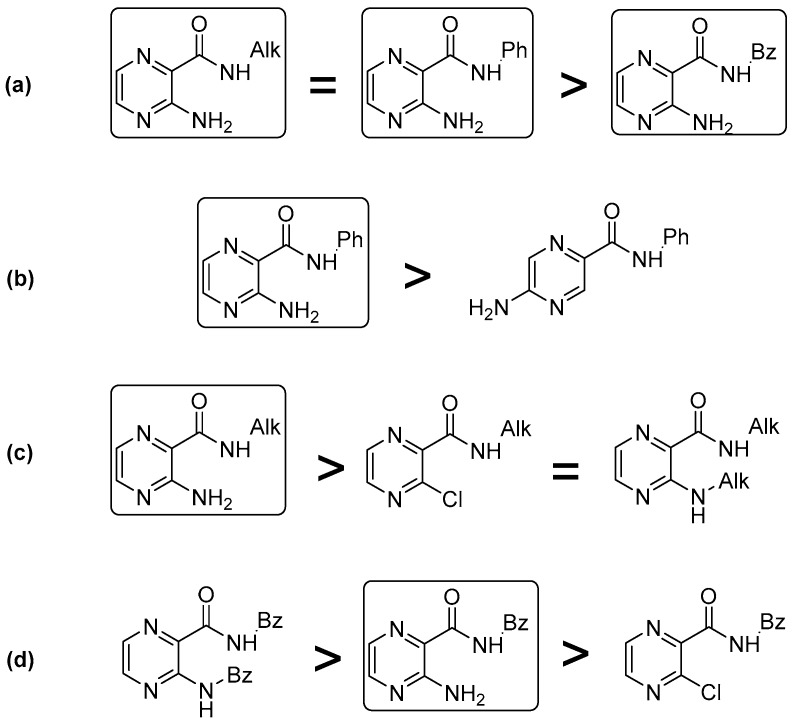
A comparison between antimycobacterial activity against *Mtb* (**a**) between the three structural subtypes of the current series (**b**) between phenyl derivatives belonging to this series and their positional isomers [16] (**c**) among different alkyl derivatives [14] (**d**) among different benzyl derivatives [15]. Structures in rounded rectangles belong to the current series. Alk = alkyl; Ph = phenyl; Bz = benzyl. Structures in rectangles belong to the current series.

To summarize activities against *Mtb*, phenyl and alkyl derivatives had comparable antimycobacterial activities and both were more active than benzyl derivatives (Figure 3a). Some of the phenyl derivatives exerted antimycobacterial activity against *Mtb*, while their previously prepared positional isomers with free amino group in position 5 on the pyrazine core were inactive (MIC > 100 µg/mL) [16] (see Figure 3b). Furthermore, our cumulative work indicates that the presence of a chlorine atom instead of a free amino group in position 3 on the pyrazine ring does not improve and may even lower the antimycobacterial activity of alkyl derivatives (Figure 3c), and benzyl derivatives (Figure 3d) [14]. Phenyl derivatives with chlorine atoms at position 3 have not been tested for antimycobacterial activity so far. For benzyl derivatives, substituting the free amino group in position 3 with a benzyl group improved the activity of the original compounds, however for alkyl derivatives, substituting the free amino group with alkyl chains showed no improvement in antimycobacterial activity against *Mtb* [14,15], see Figure 3c,d. 

Regarding activity against *M. smegmatis*, compound **20** (R’ = 4-CF_3_) exerted high activity (MIC = 31.25 µg/mL, 111 µM), while compounds **11** (MIC = 62.5 µg/mL, 264 µM) and **12** (MIC = 62.5 µg/mL, 250 µM) showed weaker activity. No activity was observed against *M. avium* and *M. aurum* (MIC > 100 µg/mL). 

### 2.4. Antibacterial and Antifungal Activity

In a microdilution in vitro assay, the final compounds were screened against four Gram-positive [*Staphylococcus aureus* (SA), methicillin resistant *Staphylococcus aureus* (MRSA), *Staphylococcus epidermidis* (SE), *Enterococcus faecalis* (EF)] and four Gram-negative [*Escherichia coli* (EC), *Klebsiella pneumoniae* (KP), *Serratia marcescens* (SM), *Pseudomonas aeruginosa* (PA)] bacterial strains and eight fungal strains [*Candida albicans* (CA), *Candida krusei* (CK), *Candida parapsilosis* (CP), *Candida tropicalis* (CT), *Aspergillus fumigatus* (AF), *Aspergillus flavus* (AFla), *Absidia corymbifera* (AC), *Trichophyton interdigitale* (TI)] of clinical importance. Antibacterial and antifungal activity is expressed as MIC in µM. The discussed MIC values were read after 24 h and 48 h of incubation, except for TI for which the MIC values were measured after 72 h and 120 h of incubation time. All MIC values obtained from the two time points were indifferent for each pathogen. 

Among the three subtypes of this series, benzyl derivatives **1**–**8** did not exert any antibacterial activity against the tested bacterial strains. When the methylene (-CH_2_-) linker was removed (phenyl derivatives **13**–**20**), two compounds were found to be active against staphylococcal strains. The most active phenyl derivative **20** (R’ = 4-CF_3_) exerted activity (MIC = 31.25 µM) against *Staphylococcus aureus* and weaker activity (MIC = 62.5 µM) against methicillin resistant *S. aureus* (MRSA) and *S. epidermidis*. This is in agreement with the activity of its previously published positional isomer, 5-amino-*N*-(4-(trifluoromethyl)phenyl)pyrazine-2-carboxamide, which had activity against *P. aeruginosa* (MIC = 250 µM) [16]. Compound **18** (R’ = 2,4-diF) was not tested due to solubility issues. Among the alkyl derivatives **9**–**12,** weak antibacterial activity against the tested strains was observed for compounds having alkyl chain with six carbons (compound **10**; MIC = 500 µM) and seven carbons (compound **11**; MIC = 250 µM). The antibacterial activity again increased with the prolongation of alkyl chain; compound **12** (R’ = C_8_H_17_) had the highest antibacterial activity (MIC = 62.5 µM). 

Marginal antifungal activity was found in all three structural types. Benzyl derivatives **1**, **3**, **4** and **7** showed activity mainly against *TI*. Alkyl derivatives with even number of carbons in alkyl chain (**9**, **11**) exerted lower activity (MIC_72h_ = 500 µM) than compounds with odd number (**10**, **12**) (MIC_72h_ = 250 µM) against *TI*. In the phenyl derivatives group, antifungal activity was limited to *CA* In contrast to phenyl derivatives belonging to this series, their positional isomers with free amino group in position 5 of the pyrazine ring had no antifungal activity [16]. It must be noted that the antifungal activity observed in this study was insignificant in comparison to reference drugs (cf. Section 3.5 and Section 3.6). 

### 2.5. Cytotoxicity

The most active compound against *Mtb* (compound **17**) and the compound with the broadest spectrum of activity (compound **20**) were further evaluated for *in vitro* cytotoxicity in HepG2 cell line. A hepatocellular cancer model was chosen since antitubercular drugs are known to carry the risk of hepatotoxicity that is even augmented when agents are administered in combination [30,31]. The obtained results were presented by the inhibitory concentration that reduces the viability of the cell population to 50% of the maximal viability (IC_50_). Compound **20** (R’ = 4-CF_3_) exerted moderate cytotoxicity with IC_50_ = 41.4 µM. Compound **17** showed no cytotoxicity up to the highest tested concentration (50 µM for compound **17** due to decreased solubility in the testing medium). IC_50_ values for additionally tested compounds **10** (IC_50_ = 389 µM) and **16** (IC_50_ > 250 µM) indicate that the cytotoxicity of discussed compounds can be modified (decreased) by various substitution of the carboxamide functional group. Thus, the observed cytotoxicity is not a general property of the 3-aminopyrazine-2-carboxamide moiety. 

## 3. Experimental

### 3.1. General Information

All chemicals were of reagent or higher grade of purity. They were purchased from Sigma-Aldrich (Steinheim, Germany), unless stated otherwise. Progress of reactions was monitored on Merck Silica 60 F254 TLC plates (Merck, Darmstadt, Germany) with UV detection using 254 nm wavelength. Microwave assisted reactions were performed in a CEM Discover microwave reactor with focused field (CEM Corporation, Matthews, NC, USA) connected to Explorer 24 autosampler (CEM Corporation) and this equipment was controlled by CEM’s Synergy^TM^ software. The temperature of the reaction mixture in the microwave reactor was monitored by internal infrared sensor. All obtained products were purified by preparative flash chromatograph CombiFlash^®^ Rf (Teledyne Isco Inc., Lincoln, NE, USA). The type of elution was gradient, using the mixture of hexane (LachNer, Neratovice, Czech Republic) and EtOAc (Penta, Prague, Czech Republic) as mobile phase. Silica gel (0.040–0.063 mm, Merck) was used as the stationary phase. NMR spectra were recorded on a Varian VNMR S500 instrument (Varian, Palo Alto, CA, USA) at 500 MHz for ^1^H and 125 MHz for ^13^C. Chemical shifts were reported in ppm (δ) and were referred indirectly to tetramethylsilane via signal of solvent (2.49 for ^1^H and 39.7 for ^13^C in DMSO-*d*_6_; 7.26 for ^1^H and 77.2 for ^13^C in CDCl_3_). Infrared spectra were recorded with a FT-IR Nicolet 6700 spectrometer (Thermo Scientific, Waltham, MA, USA) using attenuated total reflectance (ATR) methodology on germanium crystal. Elemental analysis was performed on vario MICRO cube Element Analyzer (Elementar Analysensysteme, Hanau, Germany). All values regarding elemental analyses are given as percentages. Melting points were determined in open capillary on Stuart SMP30 melting point apparatus (Bibby Scientific Limited, Staffordshire, UK) and are uncorrected. Yields are expressed as percentages of theoretical yields and refer to the isolated products (chromatographically pure) after all purification steps. Theoretical lipophilicity parameters log *P* and Clog*P* were calculated by CS ChemBioDraw Ultra 17.1 (CambridgeSoft, Cambridge, MA, USA). Experimental lipophilicity parameter log *k* was determined using reverse phase HPLC (see Section 3.8). R_f_ values were calculated using TLC (Merck Silica 60 F_254_) in hexane/ethyl-acetate 1:1 system.

### 3.2. Chemistry

#### 3.2.1. General Procedure A

Used for *N*-benzyl derivatives **1**–**8**. The starting 3-aminopyrazine-2-carboxylic acid (Sigma-Aldrich, Schnelldorf, Germany; 2.2 g; 15.8 mmol) in methanol (250 mL) was cooled down to 0 °C and concentrated H_2_SO_4_ (3.2 mL) was added. The reaction mixture was stirred for 48 h at rt. Subsequently the reaction mixture was poured into water (27 mL) and alkalized by NaHCO_3_ (approx. 6.3 g) to pH = 7. The precipitate was filtered off and collected as white-brown solid to obtain methyl 3-aminopryzine-2-carboxylate [32]. Methyl 3-aminopyrazine-2-carboxylate (100 mg; 0.65 mmol) in methanol (2 mL) along with substituted benzylamine (1.95 mmol; 3 equiv) and NH_4_Cl (10 mg; 0.19 mmol; 0.1 equiv) [33] were put into reaction tube used for microwave assisted synthesis with a magnetic stirrer. Reaction was performed in a CEM Discover microwave reactor with a focused field in pressurized vials with the following settings: 130 °C, 90 W and 40 min. The progress of the reaction was checked using TLC (Merck Silica 60 F_254_) developed by in hexane/ethyl-acetate 2:1 system. The reaction mixture was adsorbed to silica gel and purified by flash chromatography (Teledyne Isco Inc.,) using silica (irregular, 40–63 µm) as a stationary phase and gradient elution EtOAc in hexane 0–100%, with respect to the expected lipophilicity of the product.

#### 3.2.2. General Procedure B (Used for Compounds **1**–**2** and **5**–**20**)

The starting 3-aminopyrazine-2-carboxylic acid (200 mg; 1.44 mmol) was treated with 1,1′-carbonyldiimidazole (CDI; 303 mg; 1.88 mmol; 1.3 equiv) in DMSO (2 mL) in a special tube used for microwave reactions. The reaction mixture was left to react for 5–10 min until the bubbling due to emerging CO_2_ ceased. Subsequently the corresponding alkylamine/aniline/benzylamine (2.15 mmol, 1.5 equiv) was added. The microwave reactor was used to form the amide bond under followed conditions: 120 °C, 30 min, 100 W. The progress of the reaction was checked using TLC on silica desk developed by hexane/ethyl-acetate 2:1 system. Water (6 mL) was poured into the reaction mixture, the precipitate was filtered off, dissolved again in acetone and adsorbed to silica. The prepared sample was subjected to flash chromatography as described in previous section.

### 3.3. Analytical Data

*3-Amino-N-benzylpyrazine-2-carboxamide* (**1**). Prepared by general procedure A (yield 29%) and B (yield 75%). Pale yellow solid. M. p. 118.7–120.2 °C (literature: 118–119 °C [34]); IR (ATR-Ge, cm^−1^): 3401, 3368 (NH_2_), 3287 (NH, CONH), 2930 (CH_2_), 1645 (CO, CONH); ^1^H-NMR (DMSO-*d*_6_) δ 9.26 (t, *J* = 6.4 Hz, 1H, CONH), 8.21 (d, *J* = 2.4 Hz, 1H, pyr.), 7.82 (d, *J* = 2.3 Hz, 1H, pyr.), 7.53 (bs, 2H, NH_2_), 7.33–7.28 (m, 4H, ArH), 7.25–7.20 (m, 1H, ArH), 4.45 (d, *J* = 6.4 Hz, 2H, CH_2_); ^13^C-NMR (DMSO-*d*_6_) δ 166.11, 155.36, 146.98, 139.64, 131.08, 128.41, 127.47, 126.90, 125.88, 42.29; Elemental analysis: calc. for C_12_H_12_N_4_O (MW 228.26): 63.15% C, 5.30% H, 24.55% N; found 62.68% C, 5.67% H, 24.31% N; log *k* = 0.056; R_f_ = 0.43; CAS#22908-04-9.

*3-Amino-N-(2-methylbenzyl)pyrazine-2-carboxamide* (**2**). Prepared by general procedure A (yield 27%) and B (yield 91%). Pale yellow solid. M. p. 124.5–125.6 °C; IR (ATR-Ge, cm^−1^): 3380, 3318 (NH_2_), 3145 (NH, CONH), 2912 (CH_2_), 1661 (CO, CONH); ^1^H-NMR (DMSO-*d*_6_) δ 9.09 (t, *J* = 6.2 Hz, 1H, CONH), 8.21 (d, *J* = 2.3 Hz, 1H, pyr.), 7.83 (d, *J* = 2.4 Hz, 1H, pyr.), 7.49 (bs, 2H, NH_2_), 7.23–7.19 (m, 1H, ArH), 7.15–7.11 (m, 3H, ArH), 4.44 (d, *J* = 6.3 Hz, 2H, CH_2_), 3.34 (s, 3H, CH_3_); ^13^C-NMR (DMSO-*d*_6_) δ 166.06, 155.36, 147.01, 137.19, 135.44, 131.12, 130.03, 127.28, 126.89, 125.91, 125.88, 41.25, 18.89; Elemental analysis: calc. for C_13_H_14_N_4_O (MW 242.28): 64.45% C, 5.82% H, 23.13% N; found 64.23% C, 6.30% H, 22.79% N; log *k* = 0.278; R_f_ = 0.43; CAS#1090706-56-1.

*3-Amino-N-(4-methylbenzyl)pyrazine-2-carboxamide* (**3**). Prepared by general procedure B (yield 69%). Pale yellow solid. M. p. 111.4–112.6 °C; IR (ATR-Ge, cm^−1^): 3384, 3265 (NH_2_), 3156 (NH, CONH), 2929 (CH_2_), 1658 (CO, CONH); ^1^H-NMR (CDCl_3_) δ 8.19 (bs, 1H, CONH), 8.14 (d, *J* = 2.4 Hz, 1H, pyr.), 7.76 (d, *J* = 2.4 Hz, 1H, pyr.), 7.28–7.23 (m, 2H, ArH), 7.17 (d, *J* = 7.8 Hz, 2H, ArH), 4.58 (d, *J* = 6.0 Hz, 2H, CH_2_), 2.35 (s, 3H, CH_3_); ^13^C-NMR (CDCl_3_) δ 165.81, 155.05, 146.55, 137.20, 134.95, 131.55, 129.36, 127.70, 126.55, 42.95, 21.06; Elemental analysis: calc. for C_13_H_14_N_4_O (MW 242.28): 64.45% C, 5.82% H, 23.13% N; found 64.15% C, 6.03% H, 22.70% N; log *k* = 0.289; R_f_ = 0.42; CAS#1031489-34-5.

*3-Amino-N-(4-methoxybenzyl)pyrazine-2-carboxamide* (**4**). Prepared by general procedure B (yield 57%). Pale yellow solid. M. p. 103.9–105.4 °C; IR (ATR-Ge, cm^−1^): 3408, 3347 (NH_2_), 3293(NH, CONH), 2944 (CH_2_), 1641 (CO, CONH); ^1^H NMR (DMSO-*d*_6_) δ 9.17 (t, *J* = 6.4 Hz, 1H, CONH), 8.19 (d, *J* = 2.4 Hz, 1H, pyr.), 7.81 (d, *J* = 2.4 Hz, 1H, pyr.), 7.53 (bs, 2H, NH_2_), 7.24 (d, *J* = 8.6 Hz, 2H, ArH), 6.86 (d, *J* = 8.7 Hz, 2H, ArH), 4.37 (d, *J* = 6.4 Hz, 2H, CH_2_), 3.71 (s, 3H, CH_3_); ^13^C-NMR (DMSO) δ 165.94, 158.37, 155.34, 146.92, 131.61, 131.05, 128.92, 125.94, 113.82, 55.20, 41.73; Elemental analysis: calc. for C_13_H_14_N_4_O_2_ (MW 258.28): 60.45% C, 5.46% H, 21.69% N; found 60.34% C, 5.87% H, 21.45% N; log *k* = 0.026; R_f_ = 0.33; CAS#221539-34-0.

*3-Amino-N-(2,4-dimethoxybenzyl)pyrazine-2-carboxamide* (**5**). Prepared by general procedure A (yield 39%) and B (yield 26%). Pale brown solid. M. p. 115.4–116.8 °C; IR (ATR-Ge, cm^−1^): 3413, 3256 (NH_2_), 3155 (NH, CONH), 2939 (CH_2_), 1657 (CO, CONH); ^1^H-NMR (DMSO-*d*_6_) δ 8.80 (t, *J* = 6.3 Hz, 1H, CONH), 8.20 (d, *J* = 2.3 Hz, 1H, pyr.), 7.81 (d, *J* = 2.4 Hz, 1H, pyr.), 7.52 (bs, 2H, NH_2_), 7.07 (d, *J* = 8.3 Hz, 1H, ArH), 6.55 (d, *J* = 2.4 Hz, 1H, ArH), 6.46 (dd, *J* = 8.3, 2.4 Hz, 1H, ArH), 4.36 (d, *J* = 6.2 Hz, 2H, CH_2_), 3.81 (s, 3H, CH_3_), 3.73 (s, 3H, CH_3_); ^13^C-NMR (DMSO-*d*_6_) δ 165.85, 159.89, 157.84, 155.33, 147.00, 131.10, 128.66, 125.81, 118.86, 104.53, 98.46, 55.64, 55.36, 37.34; Elemental analysis: calc. for C_14_H_16_N_4_O (MW 288.31): 58.32% C, 5.59% H, 19.43% N; found 57.08% C, 6,08% H, 19.00% N; log *k* = 0.188; R_f_ = 0.31; CAS#1197482-00-0.

*3-Amino-N-(3,4-dichlorobenzyl)pyrazine-2-carboxamide* (**6**). Prepared by general procedure A (yield 45%) and B (yield 79%). White solid. M. p. 143.4–144.4 °C; IR (ATR-Ge, cm^−1^): 3396, 3297 (NH_2_), 3190 (NH, CONH), 2930 (CH_2_), 1670 (CO, CONH); ^1^H-NMR (DMSO-*d*_6_) δ 9.38 (t, *J* = 6.4 Hz, 1H, CONH), 8.21 (d, *J* = 2.3 Hz, 1H, pyr.), 7.83 (d, *J* = 2.3 Hz, 1H, pyr.), 7.57–7.54 (m, 4H, 2ArH, NH_2_), 7.31–7.28 (m, 1H, ArH), 4.43 (d, *J* = 6.5 Hz, 2H, CH_2_); ^13^C-NMR (DMSO-*d*_6_) δ 166.33, 155.34, 147.13, 140.97, 131.13, 130.95, 130.60, 129.51, 129.44, 127.89, 125.63, 41.36; Elemental analysis: calc. for C_12_H_10_Cl_2_N_4_O (MW 297.14): 48.51% C, 3.39% H, 18.86% N; found 48.92% C, 3.86% H, 18.62% N; log *k* = 0.493; R_f_ = 0.35.

*3-Amino-N-(3-(trifluoromethyl)benzyl)pyrazine-2-carboxamide* (**7**). Prepared by general procedure A (yield 18%) and B (yield 74%). Pale yellow solid. M. p. 82.2–84.8 °C; IR (ATR-Ge, cm^−1^): 3404, 3374 (NH_2_), 3283 (NH, CONH), 2947 (CH_2_), 1646 (CO, CONH); ^1^H-NMR (DMSO-*d*_6_) δ 9.43 (t, *J* = 6.4 Hz, 1H, CONH), 8.21 (d, *J* = 2.4 Hz, 1H, pyr.), 7.83 (d, *J* = 2.4 Hz, 1H, pyr.), 7.67 (d, *J* = 2.1 Hz, 1H, ArH), 7.64–7.52 (m, 5H, 3ArH, NH_2_), 4.53 (d, *J* = 6.4 Hz, 2H, CH_2_); ^13^C-NMR (DMSO-*d*_6_) δ 166.64, 155.64, 147.40, 141.50, 131.96, 131.42, 129.78, 129.42 (q, *J* = 31.7 Hz) 125.97, 124.96 (q, *J* = 272.0 Hz), 124.33 (q, *J* = 3.8 Hz), 123.96 (q, *J* = 3.7 Hz), 42.25; Elemental analysis: calc. for C_13_H_11_F_3_N_4_O (MW 296.25): 52.71% C, 3.74% H, 18.91% N; found 52.39% C, 3.64% H, 18.74% N; log *k* = 0.282; R_f_ = 0.37; CAS#1090354-24-7.

*3-Amino-N-(4-(trifluoromethyl)benzyl)pyrazine-2-carboxamide* (**8**). Prepared by general procedure A (yield 16%) and B (yield 55%). Pale yellow solid. M. p. 117.9–120.1 °C; IR (ATR-Ge, cm^−1^): 3413, 3286 (NH_2_), 3196 (NH, CONH), 2943 (CH_2_), 1650 (CO, CONH); ^1^H-NMR (DMSO-*d*_6_) δ 9.42 (t, *J* = 6.4 Hz, 1H, CONH), 8.22 (d, *J* = 2.3 Hz, 1H, pyr.), 7.84 (d, *J* = 2.4 Hz, 1H, pyr.), 7.66 (d, *J* = 8.1 Hz, 2H, ArH), 7.52 (d, *J* = 8.0 Hz, 4H, 2ArH, NH_2_), 4.53 (d, *J* = 6.4 Hz, 2H, CH_2_); ^13^C-NMR (DMSO-*d*_6_) δ 166.39, 155.38, 147.13, 144.58, 131.15, 128.09, 127.63 (q, *J* = 31.6 Hz), 125.71, 125.31 (q, *J* = 3.8 Hz), 124.52 (q, *J* = 271.6 Hz), 42.04; Elemental analysis: calc. for C_13_H_11_F_3_N_4_O (MW 296.25): 52.71% C, 3.74% H, 18.91% N; found 53.05% C, 4.12% H, 18.56% N; log *k* = 0.308; R_f_ = 0.36; CAS#1210409-77-0.

*3-Amino-N-pentylpyrazine-2-carboxamide* (**9**). Prepared by general procedure B (yield 67%). Pale yellow solid. M. p. 58.1–60.3 °C; IR (ATR-Ge, cm^−1^): 3406, 3310 (NH_2_), 3035 (NH, CONH), 2955 (CH_3_), 2928, 2856 (CH_2_), 1669 (CO, CONH); ^1^H-NMR (CDCl_3_) δ 8.13 (d, *J* = 2.4 Hz, 1H, pyr.), 7.91 (bs, 1H, CONH), 7.77 (d, *J* = 2.3 Hz, 1H, pyr.), 3.43–3.38 (m, 2H, CH_2_), 1.65–1.58 (m, 2H, CH_2_), 1.39–1.34 (m, 4H, CH_2_), 0.91 (t, *J* = 7.0 Hz, 3H, CH_3_); ^13^C-NMR (CDCl_3_) δ 165.85, 155.00, 146.35, 131.44, 126.76, 39.15, 29.26, 29.09, 22.33, 13.94; Elemental analysis: calculated for C_10_H_16_N_4_O (208.27): 57.67% C; 7.74% H; 26.90% N; found 57.17% C; 7.35% H; 26.48% N; log *k* = 0.327; R_f_ = 0.52; CAS#1090413-66-3.

*3-Amino-N-hexylpyrazine-2- carboxamide* (**10**). Prepared by general procedure B (yield 50%). Yellow solid. M. p. 30.1–32.1 °C; IR (ATR-Ge, cm^−1^): 3389, 3255 (NH_2_), 3153 (NH, CONH), 2958 (CH_3_), 2937, 2870, 2857 (CH_2_), 1660 (CO, CONH); ^1^H-NMR (CDCl_3_) δ 8.11 (d, *J* = 2.4 Hz, 1H, pyr.), 7.90 (bs, 1H, CONH), 7.77 (d, *J* = 2.2 Hz, 1H, pyr.), 3.43–3.37 (m, 2H, CH_2_), 1.65–1.56 (m, 2H, CH_2_), 1.41–1.34 (m, 2H, CH_2_), 1.34–1.26 (m, 4H, CH_2_), 0.89 (t, *J* = 7.1 Hz, 3H, CH_3_); ^13^C-NMR (CDCl_3_) δ 165.80, 154.93, 146.12, 131.38, 126.88, 39.18, 31.43, 29.52, 26.60, 22.49, 13.95; Elemental analysis: calculated for C_11_H_18_N_4_O (222.29): 59.44% C; 8.16% H; 25.20% N; found 58.98% C; 8.38% H; 25.79% N; log *k* = 0.569; R_f_ = 0.57; CAS#1090592-99-6.

*3-Amino-N-heptylpyrazine-2-carboxamide* (**11**). Prepared by general procedure B (yield 42%). Yellow solid. M. p. 33.5–35.4 °C; IR (ATR-Ge, cm^−1^): 3394, 3254 (NH_2_), 3152 (NH, CONH), 2951 (CH_3_), 2936, 2916, 2855 (CH_2_), 1663 (CO, CONH); ^1^H-NMR (CDCl_3_) δ 8.09 (d, *J* = 2.5 Hz, 1H, pyr.), 7.88 (t, *J* = 6.5 Hz, 1H, CONH), 7.77 (d, *J* = 2.4 Hz, 1H, pyr.), 3.42–3.37 (m, 2H, CH_2_), 1.63–1.57 (m, 2H, CH_2_), 1.40–1.23 (m, 8H, CH_2_), 0.87 (t, *J* = 6.9 Hz, 3H, CH_3_); ^13^C-NMR (CDCl_3_) δ 165.57, 154.53, 145.07, 131.21, 127.41, 39.21, 31.66, 29.53, 28.90, 26.89, 22.52, 13.99; Elemental analysis: calculated for C_12_H_20_N_4_O (236.32): 60.99% C; 8.53% H; 23.71% N; found 60.52% C; 8.75% H; 23.39% N; log *k* = 0.813; R_f_ = 0.61.

*3-Amino-N-octylpyrazine-2-carboxamide* (**12**). Prepared by general procedure B (yield 51%). Yellow solid. M. p. 40.3–40.9 °C; IR (ATR-Ge, cm^−1^): 3394, 3363 (NH_2_), 3275 (NH, CONH), 2957 (CH_3_), 2925, 2851 (CH_2_), 1650 (CO, CONH); ^1^H-NMR (CDCl_3_) δ 8.09 (d, *J* = 2.5 Hz, 1H, pyr.), 7.88 (bs, 1H, CONH), 7.79 (d, *J* = 2.5 Hz, 1H, pyr.), 3.42–3.37 (m, 2H, CH_2_), 1.64–1.57 (m, 2H, CH_2_), 1.40–1.22 (m, 10H, CH_2_), 0.86 (t, *J* = 7.1 Hz, 3H, CH_3_); ^13^C-NMR (CDCl_3_) δ 165.41, 154.21, 144.34, 131.14, 127.80, 39.23, 31.72, 29.51, 29.19, 29.12, 26.92, 22.56, 14.01; Elemental analysis: calculated for C_13_H_22_N_4_O (250.35): 62.37% C; 8.86% H; 22.38% N; found 61.91% C; 9.32% H; 21.97% N; log *k* = 1.057; R_f_ = 0.64. 

*3-Amino-N-phenylpyrazine-2-carboxamide* (**13**). Prepared by general procedure B (yield 43%). Pale beige solid. M. p. 105.4–106.8 °C (literature: 106–107 °C [18]); IR (ATR-Ge, cm^−1^): 3400, 3338 (NH_2_), 3266 (NH, CONH), 1671 (CO, CONH); ^1^H-NMR (CDCl_3_) δ 9.81 (s, 1H, CONH), 8.20 (d, *J* = 2.4 Hz, 1H, pyr.), 7.86 (d, *J* = 2.3 Hz, 1H, pyr.), 7.73–7.68 (m, 2H, ArH), 7.42–7.36 (m, 2H, ArH), 7.19–7.14 (m, 1H, ArH); ^13^C-NMR (CDCl_3_) δ 163.89, 155.32, 147.02, 137.40, 131.48, 129.04, 126.15, 124.39, 119.76; Elemental analysis: calculated for C_11_H_10_N_4_O (214.23): 61.67% C; 4.71% H; 26.15% N; found 62.01% C; 4.79% H; 25.99% N; log *k* = 0.189; R_f_ = 0.51; CAS#36204-80-5.

*3-Amino-N-(4-hydroxyphenyl)pyrazine-2-carboxamide* (**14**). Prepared by general procedure B (yield 55%). Yellow solid. M. p. 224.0–226.2 °C; IR (ATR-Ge, cm^−1^): 3477, 3348 (NH_2_), 3024 (NH, CONH), 1665 (CO, CONH); ^1^H-NMR (DMSO-*d*_6_) δ 10.27 (s, 1H, CONH), 9.28 (s, 1H, OH), 8.24 (d, *J* = 2.4 Hz, 1H, pyr.), 7.88 (d, *J* = 2.3 Hz, 1H, pyr.), 7.61–7.56 (m, 4H, 2ArH, NH_2_), 6.76–6.71 (m, 2H, ArH); ^13^C-NMR (DMSO-*d*_6_) δ 164.15, 155.56, 154.10, 147.17, 131.04, 129.92, 125.95, 122.40, 115.21; Elemental analysis: calculated for C_11_H_10_N_4_O_2_ (230.23): 57.39% C; 4.38% H; 24.34% N; found 57.34% C; 4.32% H; 24.12% N; log *k* = 0.382; R_f_ = 0.33.

*3-Amino-N-(2,5-dimethylphenyl)pyrazine-2-carboxamide* (**15**). Prepared by general procedure B (yield 21%). Pale yellow solid. M. p. 212.5–213.2 °C; IR (ATR-Ge, cm^−1^): 3416, 3331 (NH_2_), 3293 (NH, CONH), 3052 (CH_3_), 1675 (CO, CONH); ^1^H-NMR (DMSO-*d*_6_) δ 10.04 (s, 1H, CONH), 8.28 (d, *J* = 2.3 Hz, 1H, pyr.), 7.90 (d, *J* = 2.3 Hz, 1H, pyr.), 7.59 (s, 3H, 1ArH, NH_2_), 7.12 (d, *J* = 7.7 Hz, 1H, ArH), 6.91 (d, *J* = 7.6 Hz, 1H, ArH), 2.27 (s, 3H, CH_3_), 2.21 (s, 3H, CH_3_); ^13^C-NMR (DMSO-*d*_6_) δ 164.29, 155.62, 147.63, 135.73, 135.42, 131.21, 130.26, 127.52, 125.83, 125.35, 124.01, 20.91, 17.26; Elemental analysis: calculated for C_13_H_14_N_4_O (242.28): 64.45% C; 5.82% H; 23.13% N; found 63.98% C; 5.51% H; 13.06% N; log *k* = 0.778; R_f_ = 0.44.

3*-Amino-N-(4-ethylphenyl)pyrazine-2-carboxamide* (**16**). Prepared by general procedure B (yield 48%). Yellow solid. M. p. 135.0–137.4 °C; IR (ATR-Ge, cm^−1^): 3397, 3324 (NH_2_), 3264 (NH, CONH), 2960 (CH_3_), 2928, 2873 (CH_2_), 1667 (CO, CONH); ^1^H-NMR (CDCl_3_) δ 9.76 (s, 1H, CONH), 8.19 (d, *J* = 2.4 Hz, 1H, pyr.), 7.85 (d, *J* = 2.3 Hz, 1H, pyr.), 7.62–7.59 (m, 2H, ArH), 7.24–7.20 (m, 2H, ArH), 2.65 (q, *J* = 7.6 Hz, 2H, CH_2_), 1.25 (t, *J* = 7.6 Hz, 3H, CH_3_); ^13^C-NMR (CDCl_3_) δ 163.75, 155.30, 146.86, 140.48, 135.02, 131.42, 128.34, 126.31, 119.87, 28.30, 15.58; Elemental analysis: calculated for C_13_H_14_N_4_O (242.28): 64.45% C; 5.82% H; 23.13% N; found 64.50% C; 5.82% H; 22.99% N; log *k* = 0.623; R_f_ = 0.52; CAS#1031153-53-3.

*3-Amino-N-(2,4-dimethoxyphenyl)pyrazine-2-carboxamide* (**17**). Prepared by general procedure B (yield 34%). Yellow solid. M. p. 158.2–158.7 °C; IR (ATR-Ge, cm^−1^): 3364 (NH_2_), 3296 (NH, CONH), 3956, 2836 (CH_3_), 1665 (CO, CONH); ^1^H-NMR (DMSO-*d*_6_) δ 10.06 (s, 1H, CONH), 8.28 (d, *J* = 2.3 Hz, 1H, pyr.), 8.19 (d, *J* = 8.8 Hz, 1H, ArH), 7.89 (d, *J* = 2.3 Hz, 1H, pyr.), 7.62 (s, 2H, NH_2_), 6.68 (d, *J* = 2.7 Hz, 1H, ArH), 6.57–6.52 (m, 1H, ArH), 3.89 (s, 3H, CH_3_), 3.76 (s, 3H, CH_3_); ^13^C-NMR (DMSO-*d*_6_) δ 163.35, 156.58, 155.54, 150.06, 147.71, 131.24, 125.14, 120.31, 120.22, 104.39, 99.02, 56.25, 55.51; Elemental analysis: calculated for C_13_H_14_N_4_O_3_ (274.28): 56.93% C; 5.15% H; 20.43% N; found 57.36% C; 5.62% H; 20.01% N; log *k* = 0.37; R_f_ = 0.41; CAS#1320086-09-6.

*3-Amino-N-(2,4-difluorophenyl)pyrazine-2-carboxamide* (**18**). Prepared by general procedure B (yield 26%). Pale yellow solid. M. p. 186.6–188.1 °C; IR (ATR-Ge cm^−1^): 3423, 3334 (NH_2_), 3265 (NH, CONH), 1684 (CO, CONH); ^1^H-NMR (DMSO-*d*_6_) δ 10.25 (s, 1H, CONH), 8.30 (d, *J* = 2.3 Hz, 1H, pyr.), 7.91 (d, *J* = 2.3 Hz, 1H, pyr.), 7.90–7.84 (m, 1H, ArH), 7.58 (bs, 2H, NH_2_), 7.39–7.32 (m, 1H, ArH), 7.14–7.07 (m, 1H, ArH); ^13^C-NMR (DMSO-*d*_6_) δ 164.68, 159.17 (dd, *J* = 244.2, 11.6 Hz), 155.60, 154.90 (dd, *J* = 248.3, 12.6 Hz), 148.03, 131.35, 126.05 (dd, *J* = 9.6, 2.7 Hz), 124.60, 122.23 (dd, *J* = 11.5, 3.7 Hz), 111.39 (dd, *J* = 22.0, 3.6 Hz), 104.39 (dd, *J* = 26.9, 24.1 Hz); Elemental analysis: calculated for C_11_H_8_F_2_N_4_O (250.21): 52.80% C; 3.22% H; 22.39% N; found 52.56% C; 3.07% H; 21.98% N; log *k* = 0.422; R_f_ = 0.61; CAS#1334647-58-3.

*3-Amino-N-(3,4-dichlorophenyl)pyrazine-2-carboxamide* (**19**). Prepared by general procedure B (yield 26%). Pale yellow solid. M. p. 195.1–197.5 °C; IR (ATR-Ge, cm^−1^): 3417, 3338 (NH_2_), 3272 (NH, CONH), 1672 (CO, CONH); ^1^H-NMR (DMSO-*d*_6_) δ 10.79 (s, 1H, CONH), 8.29 (d, *J* = 2.3 Hz, 1H, pyr.), 8.24 (d, *J* = 2.5 Hz, 1H, ArH), 7.92 (d, *J* = 2.3 Hz, 1H, pyr.), 7.84 (dd, *J* = 8.6, 2.5 Hz, 3H, 1ArH, NH_2_), 7.58 (d, *J* = 8.8 Hz, 1H, ArH); ^13^C-NMR (DMSO-*d*_6_) δ 165.13, 155.63, 147.86, 138.61, 131.17, 130.58, 125.45, 124.92, 121.81, 120.67, 119.76; Elemental analysis: calculated for C_11_H_8_Cl_2_N_4_O (283.11): 46.67% C; 2.85% H; 19.79% N; found 47.15% C; 3.29% H; 19.32% N; log *k* = 0.778; R_f_ = 0.43.

*3-Amino-N-(4-(trifluoromethyl)phenyl)pyrazine-2-carboxamide* (**20**). Prepared by general procedure B (yield 25%). Pale yellow solid. M. p. 132.6–135.1 °C; IR (ATR-Ge, cm^−1^): 3401, 3333 (NH_2_), 3261 (NH, CONH), 1677 (CO, CONH); ^1^H-NMR (DMSO-*d*_6_) δ 10.85 (s, 1H, CONH), 8.37 (t, *J* = 2.0 Hz, 1H, ArH), 8.30 (d, *J* = 2.3 Hz, 1H, pyr.), 8.11–8.08 (m, 1H, ArH), 7.93 (d, *J* = 2.3 Hz, 1H, pyr.), 7.61 (bs, 2H, NH_2_), 7.57 (t, *J* = 8.1 Hz, 1H, ArH), 7.45–7.42 (m, 1H, ArH); ^13^C-NMR (DMSO-*d*_6_) δ 165.25, 155.68, 147.83, 139.27, 131.16, 129.91, 129.40 (q, *J* = 31.4 Hz), 125.05, 124.33 (q, *J* = 272.1 Hz), 124.25, 120.25 (q, *J* = 3.9 Hz), 116.75 (q, *J* = 4.0 Hz); Elemental analysis: calculated for C_12_H_9_F_3_N_4_O (282.23): 51.07% C; 3.21% H; 19.85% N; found 50.75% C; 3.05% H; 19.44% N; log *k* = 0.525; R_f_ = 0.51.

### 3.4. Evaluation of Antimycobacterial Activity

#### 3.4.1. Mycobacterium tuberculosis, Mycobacterium kansasii, and Mycobacterium avium

A 96-well plate microdilution broth method was performed. Tested strains *Mycobacterium tuberculosis* H37Rv CNCTC My 331/88 (ATCC 27294), *M. kansasii* CNCTC My 235/80 (ATCC 12478) and *M. avium* ssp. *avium* CNCTC My 80/72 (ATCC 15769) were obtained from the Czech National Collection of Type Cultures (CNCTC), National Institute of Public Health (Prague, Czech Republic). Middlebrook 7H9 broth of declared pH = 6.6 (Sigma-Aldrich) enriched with 0.4% of glycerol (Sigma-Aldrich) and 10% of OADC growth supplement (oleic acid, albumin, dextrose, catalase; Himedia, Mumbai, India) was used for cultivation. Tested compounds were dissolved and diluted in DMSO and mixed with broth (25 μL of DMSO solution in 4.475 mL of broth) and placed (100 μL) into microplate wells. Mycobacterial inocula were suspended in isotonic saline solution and the density was adjusted to 0.5–1.0 according to McFarland scale. These suspensions were diluted by 10^−1^ and used to inoculate the testing wells, adding 100 μL of mycobacterial suspension per well. Final concentrations of tested compounds in wells were 100, 50, 25, 12.5, 6.25, 3.13 and 1.56 μg/mL. INH was used as positive control (inhibition of growth). Negative control (visible growth) consisted of broth plus mycobacterial suspension plus DMSO (purity of broth). A total of 30 μL of Alamar Blue working solution (1:1 mixture of 0.01% resazurin sodium salt (aq. sol.) and 10% Tween 80) was added after five days of incubation. Results were then determined after 24 h of incubation. The MIC (in μg/mL) was determined as the lowest concentration that prevented the blue to pink color change. MIC values of INH were 6.25–12.5 μg/mL against *M. avium*, 3.13–12.5 μg/mL against *M. kansasii*, and 0.1–0.2 μg/mL against *M. tuberculosis* H37Rv. All experiments were conducted in duplicates.

#### 3.4.2. Mycobacterium smegmatis and Mycobacterium aurum

Antimycobacterial assay was performed with fast growing *Mycobacterium smegmatis* DSM 43465 (ATCC 607) and *Mycobacterium aurum* DSM 43999 (ATCC 23366) from German Collection of Microorganisms and Cell Cultures (Braunschweig, Germany). The technique used for activity determination was microdilution broth panel method using 96-well microtitration plates. Culturing medium was Middlebrook 7H9 broth (Sigma-Aldrich) enriched with 0.4% of glycerol (Sigma-Aldrich) and 10% of Middlebrook OADC growth supplement (Himedia, Mumbai, India). Mycobacterial strains were cultured on Middlebrook 7H10 agar and suspensions were prepared in Middlebrook 7H9 broth. Final density was adjusted to value ranging from 0.5 to 1.0 according to McFarland scale and diluted in ratio 1:20 with broth. Tested compounds were dissolved in DMSO (Sigma-Aldrich) then Middlebrook broth was added to obtain concentration 2000 µg/mL. Standards used for activity determination were isoniazid (INH), rifampicin (RIF) and ciprofloxacin (CPX) (Sigma-Aldrich). Final concentrations were reached by binary dilution and addition of mycobacterial suspension and were set as 500, 250, 125, 62.5, 31.25, 15.625, 7.81 and 3.91 µg/mL except to standards rifampicin, where the final concentrations were 12.5, 6.25, 3.125, 1.56, 0.78, 0.39, 0.195 and 0.098 µg/mL, and ciprofloxacin, where the final concentrations were 1, 0.5, 0.25, 0.125, 0.0625, 0.0313, 0.0156, 0.0078 µg/mL. The final concentration of DMSO did not exceeded 2.5% (*v*/*v*) and did not affect the growth of *M. smegmatis* nor *M. aurum*. Positive (broth, DMSO, bacteria) and negative (broth, DMSO) controls were included. Plates were sealed with polyester adhesive film and incubated in dark at 37 °C without agitation. The addition of 0.01% solution of resazurin sodium salt followed after 48 hours of incubation for *M. smegmatis*, resp. after 72 hours of incubation for *M. aurum*. Stain was prepared by dissolving resazurin sodium salt (Sigma-Aldrich) in deionised water to get 0.02% solution. Then 10% aqueous solution of Tween 80 (Sigma-Aldrich) was prepared. Both liquids were mixed up making use of the same volumes and filtered through syringe membrane filter. Microtitration panels were then incubated for further 2.5 hours for determination of activity against *M. smegmatis*, respectively 4 hours for *M. aurum*. Antimycobacterial activity was expressed as MIC and the value was read on the basis of stain colour change (blue colour – no growth; pink colour – growth). MIC values for standards were in ranges 7.81–15.625 µg/mL for INH, 12.5–25 µg/mL for RIF and 0.0625–0.125 µg/mL for CPX against *M. smegmatis*, 1.95–3.91 µg/mL for INH, 0.78–1.56 µg/mL for RIF and 0.00781–0.01563 µg/mL for CPX against *M. aurum*, respectively. All experiments were conducted in duplicates.

### 3.5. Evaluation of Antibacterial Activity

Microdilution broth method [35]. Tested strains from the Czech Collection of Microorganisms (CCM, Brno, Czech Republic) - *Staphylococcus aureus* CCM 4223 (ATCC 29213), *Staphylococcus aureus* methicillin resistant CCM 4750 (ATCC 43300), *Enterococcus faecalis* CCM 4224 (ATCC 29212), *Escherichia coli* CCM 3954 (ATCC 25922), *Pseudomonas aeruginosa* CCM 3955 (ATCC 27853). Clinical isolates from the Department of Clinical Microbiology, University Hospital in Hradec Králové, Czech Republic - *Staphylococcus epidermidis* 112-2016, *Klebsiella pneumoniae* 64-2016, *Serratia marcescens* 62-2016. All strains were subcultured on Mueller-Hinton agar (MHA) (Difco/Becton Dickinson, Detroit, MI, USA) at 35 °C and maintained on the same medium at 4 °C. The compounds were dissolved in DMSO, and the antibacterial activity was determined in cation adjusted Mueller-Hinton liquid broth (Difco/Becton Dickinson) buffered to pH 7.0. Positive controls consisted of test microbe solely, while negative controls consisted of cultivation medium and DMSO. The final concentration of DMSO in the testing medium did not exceed 1% (*v*/*v*) of the total solution composition. MIC was determined after 24 and 48 h of static incubation at 35 °C by visual inspection or using Alamar Blue dye. The standards were gentamicin and ciprofloxacin. The standards were gentamicin [MIC against *Staphylococcus aureus* 1 µg/mL (48 h); *Staphylococcus aureus methicillin resistant* 16–32 µg/mL (48 h); *Enterococcus faecalis* 8 µg/mL (48 h); *Escherichia coli* 1–2 µg/mL (48 h); *Pseudomonas aeruginosa* 0.5 µg/mL (48 h); *Staphylococcus epidermidis* >8 µg/mL (48 h); *Klebsiella pneumonia* >8 µg/mL (48 h); *Serratia marcescens* 2 µg/mL (48 h)] and ciprofloxacin [MIC against *Staphylococcus aureus* 0.128–0.256 µg/mL (48 h); *Staphylococcus aureus methicillin resistant* 0.128 µg/mL (48 h); *Enterococcus faecalis* 0.512 µg/mL (48 h); *Escherichia coli* 0.008 µg/mL (48 h); *Pseudomonas aeruginosa* 0.128 µg/mL (48 h); *Staphylococcus epidermidis* >1.024 µg/mL (48 h); *Klebsiella pneumonia* >1.024 µg/mL (48 h); *Serratia marcescens* 0.256 µg/mL (48 h)]. All experiments were conducted in duplicates. For the results to be valid, the difference in MIC for one compound determined from two parallel measurements must not be greater than one step on the dilution scale.

### 3.6. Evaluation of Antifungal Activity

Microdilution broth method [36,37]. Tested strains from the Czech Collection of Microorganisms (CCM) - *Candida albicans* CCM 8320 (ATCC 24433), *C. krusei* CCM 8271 (ATCC 6258), *C. parapsilosis* CCM 8260 (ATCC 22019), *C. tropicalis* CCM 8264 (ATCC 750), *Aspergillus flavus* CCM 8363, *Lichtheimia corymbifera* CCM 8077 and *Trichophyton interdigitale* CCM 8377 (ATCC 9533); or from the American Type Collection Cultures (ATCC, Mannasas, VA, USA)-*Aspergillus fumigatus* ATCC 204305. Compounds were dissolved in DMSO and diluted in a twofold manner with RPMI 1640 medium, with glutamine and 2% glucose, buffered to pH 7.0 with MOPS (3-morpholinopropane-1-sulfonic acid). The final concentration of DMSO in the testing medium did not exceed 1% (*v*/*v*) of the total solution composition. Static incubation was performed in the dark and in humid atmosphere, at 35 °C, for 24 and 48 h (72 and 120 h for *Trichophyton interdigitale* respectively). Positive controls consisted of test microbe solely, while negative controls consisted of cultivation medium and DMSO. MIC was inspected visually or making use of Alamar Blue indication. The standards were amphotericin B [MIC against *Candida albicans* 0.5 µg/mL (48 h); *C. krusei* 1 µg/mL (48 h); *C. parapsilosis* 0.5 µg/mL (48 h); *C. tropicalis* 1 µg/mL (48 h); *Aspergillus flavus* 8 µg/mL (48 h); *Lichtheimia corymbifera* 0.5 µg/mL (48 h); *Trichophyton interdigitale* 2 µg/mL (72 h); *Aspergillus fumigatus* 1 µg/mL (48 h)] and voriconazole [MIC against *Candida albicans* >16 µg/mL (48 h); *C. krusei* 0.5 µg/mL (48 h); *C. parapsilosis* 8 µg/mL (48 h); *C. tropicalis* >16 µg/mL (48 h); *Aspergillus flavus* >16 µg/mL (48 h); *Lichtheimia corymbifera* >16 µg/mL (48 h); *Trichophyton interdigitale* >16 µg/mL (72 h); *Aspergillus fumigatus* 1 µg/mL (48 h)]. All experiments were conducted in duplicates. For the results to be valid, the difference in MIC for one compound determined from two parallel measurements must not be greater than one step on the dilution scale.

### 3.7. Evaluation of Cytotoxicity

The human liver hepatocellular carcinoma cell line HepG2 (passage 12–13 and 17–18 for compound 20, respectively), purchased from Health Protection Agency Culture Collections (ECACC, Salisbury, UK), was routinely cultured in Minimum Essential Eagle Medium MEM (Sigma-Aldrich) supplemented with 10% fetal bovine serum (PAA Laboratories GmbH, Pasching, Austria), 2 mM l-glutamine solution (Sigma-Aldrich), and 1% non-essential amino acid solution (Sigma-Aldrich), in a humidified atmosphere containing 5% CO_2_ at 37 °C. For subculturing, the cells were harvested after trypsin/EDTA (Sigma-Aldrich) treatment at 37 °C. To evaluate the cytotoxicity, the HepG2 cells treated with the tested substances were used as experimental groups, whereas untreated HepG2 cells served as control groups. The HepG2 cells were seeded in a density of 1 × 10^4^ cells per well on a 96-well plate. The following day (24 h after seeding), they were treated with tested substances dissolved in DMSO (maximal incubation concentration of DMSO was 1%). The tested substances were prepared according to their solubility in DMSO, at incubation concentrations of 1–750 μM. The treatment was carried out in a humidified atmosphere containing 5% CO_2_ at 37 °C, in triplicate, for 24 h. The controls representing 100% cell viability, 0% cell viability (the cells treated with 10% DMSO), no-cell controls, and vehiculum controls were incubated in triplicate, simultaneously. After 24 h exposure, the reagent from the kit CellTiter 96^®^ Aqueous One Solution Cell Proliferation Assay (Promega, Madison, WI, USA) was added, according to the recommendation by the manufacturer. After 2 h incubation at 37 °C in humidified, 5% CO_2_ atmosphere, the absorbance was recorded at 490 nm. Inhibitory curves were constructed for each compound, plotting incubation concentrations vs. percentage of absorbance relative to untreated control. The standard toxicological parameter IC_50_ was calculated by a nonlinear regression analysis of the inhibitory curves using GraphPad Prism software (version 7, GraphPad Software, Inc., La Jolla, CA, USA).

### 3.8. Determination of Lipophilicity by HPLC (Log k)

Log *k* was determined using an Agilent Technologies 1200 SL liquid chromatograph with Diode-array Detector SL G1315C (Agilent Technologies Inc., Colorado Springs, CO, USA), with pre-column ZORBAX XDB-C18 5 µm, 4 mm × 4 mm and column ZORBAX Eclipse XDB-C18 5 µm, 4.6 mm × 250 mm (both Agilent Technologies Inc.). The mobile phase consisted of MeOH (HPLC grade, 70%) and H2O (HPLC-Milli-Q Grade, 30%). The flow rate was 1.0 mL/min, samples were injected in a volume of 20 µL, and the column temperature was 30 °C. Detection and monitoring wavelength were 210 nm and 270 nm, respectively. Retention times (R_t_) were measured in minutes. The dead time of the system (D_t_) was determined as the retention time of the KI methanolic solution. Capacity factors k for individual compounds were calculated according to the formula k = (R_t_ – D_t_)/D_t_. Capacity factor k was converted to log scale and (log *k*) and used as a measure of lipophilicity.

## 4. Conclusions

To conclude, a total of twenty *N*-substituted 3-aminopyrazine-2-carboxamides with free amino group in position 3 on the pyrazine ring were synthesized and evaluated for their antimicrobial activity, out of which seventeen compounds were new not previously described in literature (as of 23^rd^ of January, 2019). Compounds **1**, **4**, **13** were published [17,18,34] but were not evaluated for antimicrobial activity. The twenty compounds were divided into eight benzyl derivatives, four alkyl derivatives, and eight phenyl derivatives. We evaluated the prepared compounds for their antimycobacterial activity against *M. tuberculosis* H37Rv and four other nontubercular mycobacterial strains, along with antibacterial and antifungal activities. In the group of benzyl derivatives **1**–**8**, compounds with monosubstitution on the benzene core showed better activity, whereas in the phenyl derivatives group **13**–**20** disubstitution was preferred. For alkyl derivatives **9**–**12**, the antimycobacterial and antibacterial activities increase with the increase in the length of the carbon chain. This is in concordance with relationships observed in our previous series of pyrazinamide derivatives [26,27,28,29]. The most active compound against *Mtb* was compound **17** (phenyl derivative, R’ = 2,4-diOCH_3_) with MIC = 12.5 µg/mL, 46 µM. It was nontoxic against HepG2 cancer cell line, yet its decreased solubility (as indicated in the cytotoxicity assay) might be an issue in further development, requiring improvement of physico-chemical properties. Compound **20** (phenyl derivative, R’ = 4-CF_3_) exerted some activity against *Mtb* and high activity against *M. smegmatis*. It also exerted similar antibacterial activity against *S. aureus* and against methicillin resistant *S. aureus*. Extended cytotoxicity evaluations are advised to assess whether this broad activity is specific and not connected to general cytotoxic effects. Antibacterial activity was detected only for compounds belonging to the alkyl derivatives and phenyl derivatives groups. Antifungal activity was observed in all three structural subtypes. Antimicrobial activities of the compounds belonging to this series were compared to that of closely related compounds, for example positional isomers, previously prepared by our group. The MoA of prepared compounds is yet to be elucidated. However, based on precious docking studies of structurally related compounds, it can be suggested that compounds belonging to this series could be inhibitors of mycobacterial enoyl-ACP-reductase (InhA) [27]. 

## Figures and Tables

**Table 1:** Summary of achieved yields * of benzyl derivatives **1**–**8** by procedure A and B.

Cpd.	Yield [%]	
	Procedure A	Procedure B
**1**	29	75
**2**	27	91
**3**	69	-
**4**	57	-
**5**	39	26
**6**	45	79
**7**	18	74
**8**	16	55

* Isolated yields after all purification steps. In case of procedure A, the yield is relative to the amount of methyl ester of pyrazine-2-carboxylic acid.

**Table 2:** Prepared compounds, calculated partition coefficients log *P* and Clog*P* and antimycobacterial activity against *Mtb* and *M. smegmatis*.

Structure	No.	R’	log P*	ClogP *	MIC [µg/mL (µM)]	
					*Mtb*	*M. smegmatis*
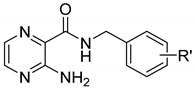	**1**	H	0.57	1.89	˃100(438.1)	˃500(2191)
	**2**	2-CH_3_	1.06	2.34	˃100(412.7)	˃500(2063.7)
	**3**	4-CH_3_	1.06	2.39	100(412.7)	˃500(2063.7)
	**4**	4-OCH_3_	0.44	1.80	100(387.2)	˃500(1935.8)
	**5**	2,4-diOCH_3_	0.32	1.90	˃100(346.8)	˃500(1734.2)
	**6**	3,4-diCl	1.69	3.20	˃100(336.5)	˃500(1682.7)
	**7**	3-CF_3_	1.49	2.77	50(168.8)	250(843.9)
	**8**	4-CF_3_	1.49	2.77	50(168.8)	˃500(1687.8)
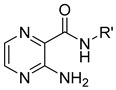	**9**	C_5_H_11_	0.50	2.04	˃100(480.1)	˃500(2400.7)
	**10**	C_6_H_13_	0.91	2.57	100(449.9)	250(1124.7)
	**11**	C_7_H_15_	1.33	3.10	50(211.6)	62.5(264.5)
	**12**	C_8_H_17_	1.75	3.63	25 (99.9)	62.5(249.7)
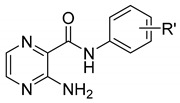	**13**	H	0.50	1.71	˃100(466.8)	˃500(2333.9)
	**14**	2-OH	0.11	1.04	˃100(434.3)	125(542.9)
	**15**	2,5-diCH_3_	1.48	2.06	˃100(412.7)	˃500(2063.7)
	**16**	4-C_2_H_5_	1.41	2.74	˃100(412.7)	˃500(2063.7)
	**17**	2,4-diOCH_3_	0.25	1.04	12.5(45.6)	˃500(1823)
	**18**	2,4-diF	0.82	1.44	˃100(399.7)	˃500(1998.3)
	**19**	3,4-diCl	1.62	3.06	50(176.6)	˃500(1766.1)
	**20**	4-CF_3_	1.42	2.65	50(176.6)	31.25(110.7)
PZA					100	˃100
INH					0.1–0.2	7.81–15.62

* Calculated by CS ChemBioDraw Ultra 17.1 (CambridgeSoft, Cambridge, MA, USA).

## Supplementary Materials

^1^H-NMR and ^13^C-NMR spectra of selected compounds from each structural subgroup [**5** (benzyl derivative), **9** (alkyl derivative), and **14** (phenyl derivative)].
