# Supplementary material for: Derivatives of 3-Aminopyrazine-2-carboxamides: Synthesis, Antimicrobial Evaluation, and in Vitro Cytotoxicity
Source: Molecules. 2019 Mar 28;24(7):1212. doi: 10.3390/molecules24071212 (PMC6479349; doi:10.3390/molecules24071212)
Supplement: Supplementary file 1 [file molecules-24-01212-s001.pdf]

Derivatives of 3-Aminopyrazine-2-carboxamides: Synthesis, Antimicrobial Evaluation, and in Vitro Cytotoxicity

Ghada Bouz [1,*], Lucia Semelková [1], Ondřej Janďourek [1], Klára Konečná [1], Pavla Paterová [2], Lucie Navrátilová [1], Vladimír Kubíček [1], Jiří Kuneš [1], Martin Doležal [1] and Jan Zitko [1,*]

[1] Faculty of Pharmacy in Hradec Králové, Charles University, Heyrovského 1203, 500 05 Hradec Králové, Czech Republic; lucia.semelkova@seznam.cz (L.S.); jando6aa@faf.cuni.cz (O.J.); konecna@faf.cuni.cz (K.K.); navratl2@faf.cuni.cz (L.N.); kubicek@faf.cuni.cz (V.K.); kunes@faf.cuni.cz (J.K.); dolezalm@faf.cuni.cz (M.D.)
[2] Department of Clinical Microbiology, Faculty Hospital, Sokolská 581, 500 05 Hradec Králové, Czech Republic; pavla.paterova@fnhk.cz
* Correspondence: bouzg@faf.cuni.cz (G.B.); jan.zitko@faf.cuni.cz (J.Z.); Tel: +420-495-067-272 (J.Z.); Fax: +420-495-518-002 (J.Z.).

Supplementary Materials

^{1}H NMR and ^{13}C NMR spectra of compound **5**

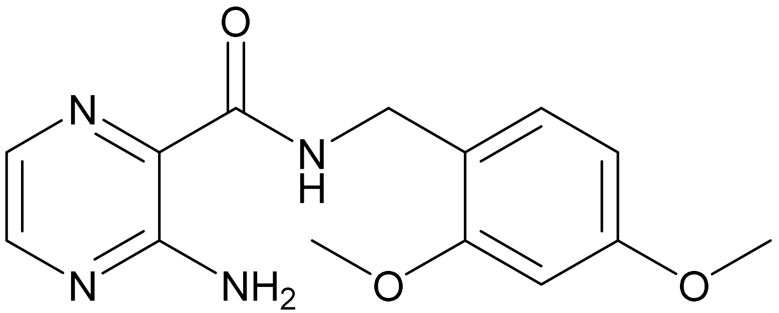

- Benzyl Group -

Compound (5)

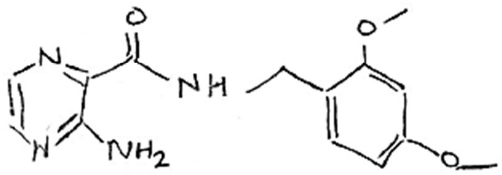

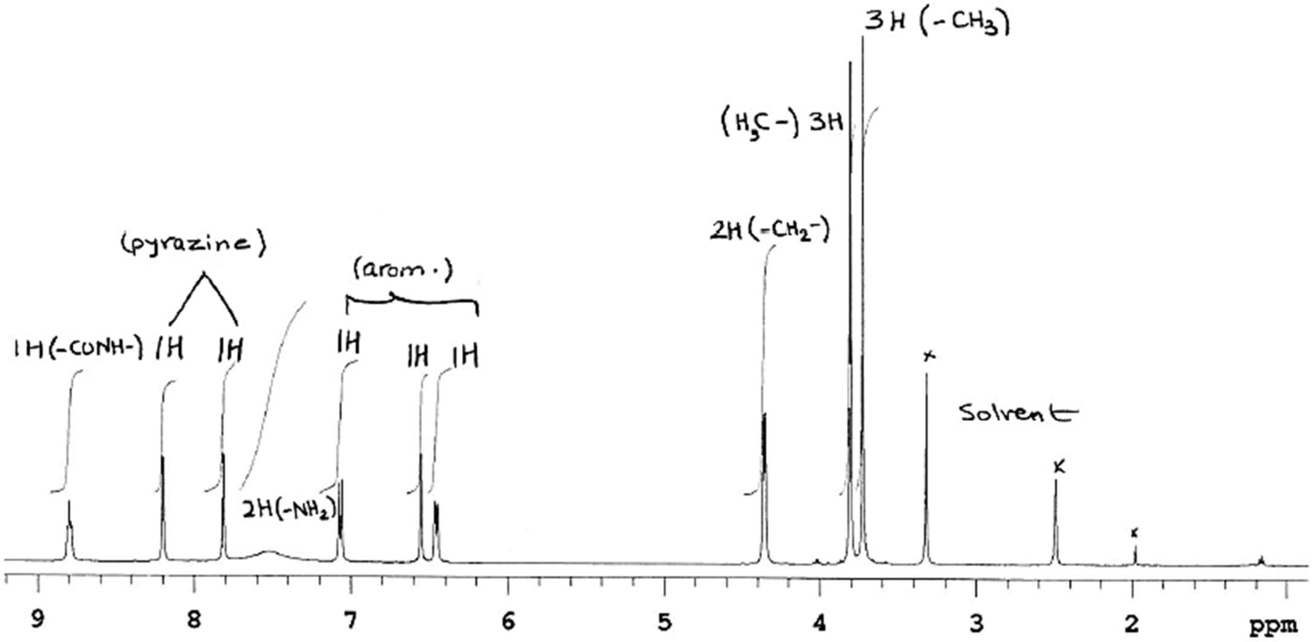

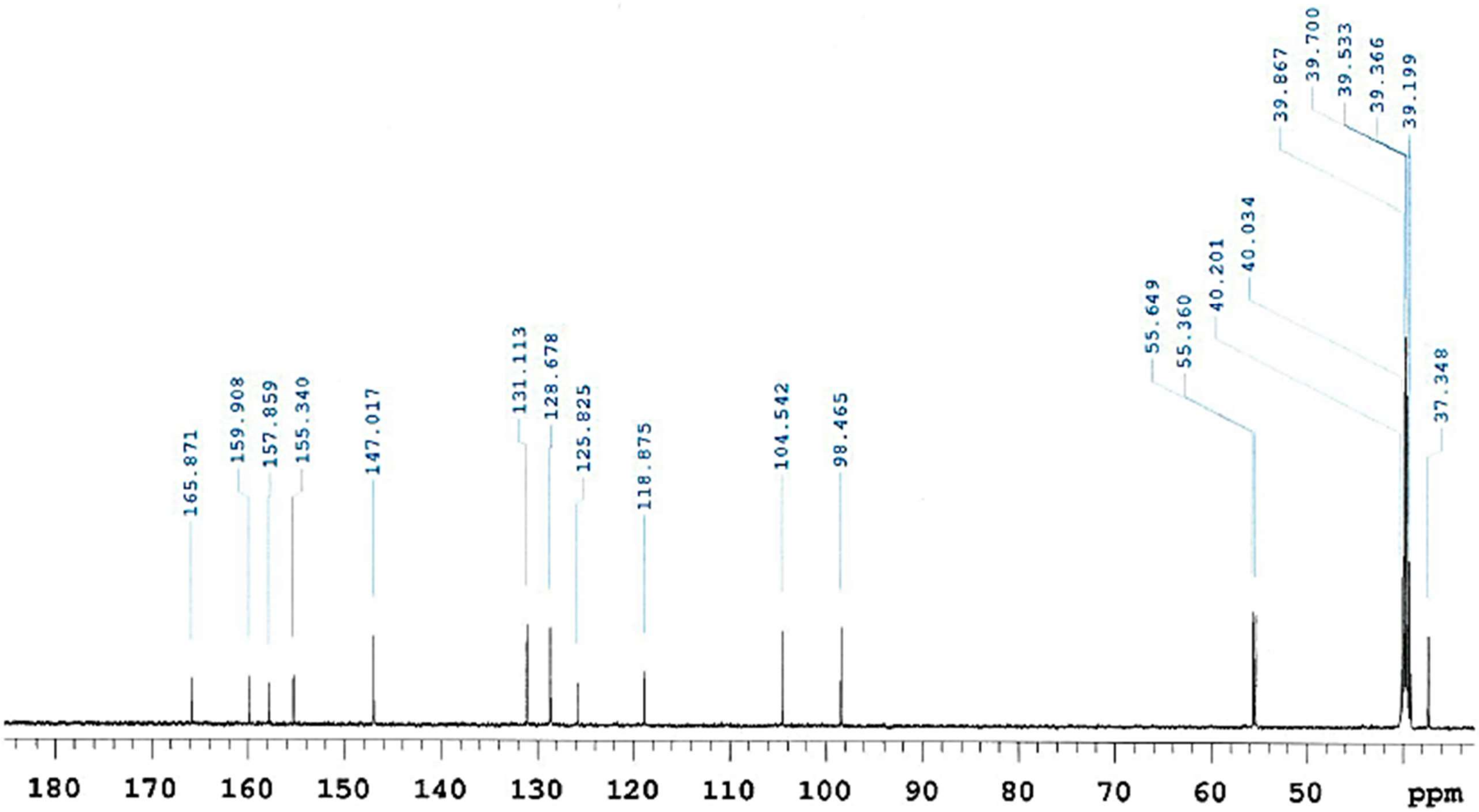

165.871
159.908
157.859
155.340
147.017
131.113
128.678
125.825
118.875
104.542
98.465
55.649
55.360
40.201
40.034
39.867
39.700
39.533
39.366
39.199
37.348
180
170
160
150
140
130
120
110
100
90
80
70
60
50
ppm

^{1}H NMR and ^{13}C NMR spectra of compound **9**

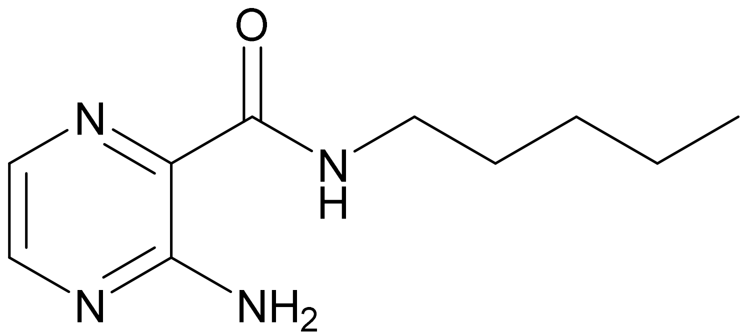

Alkyl Group-

Compound (9)

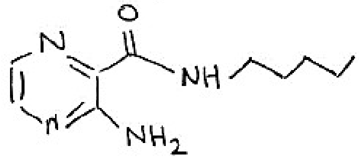

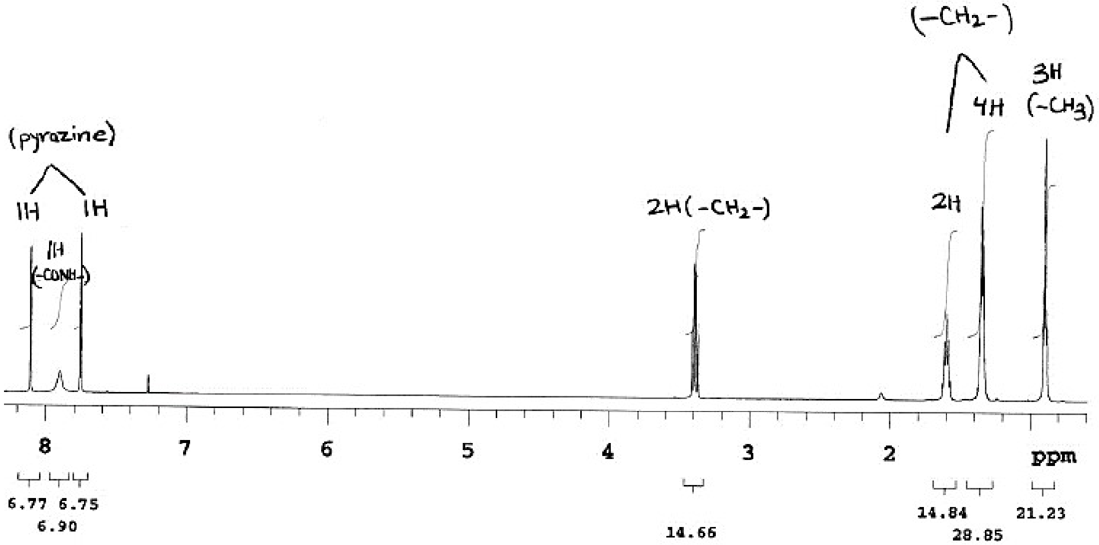

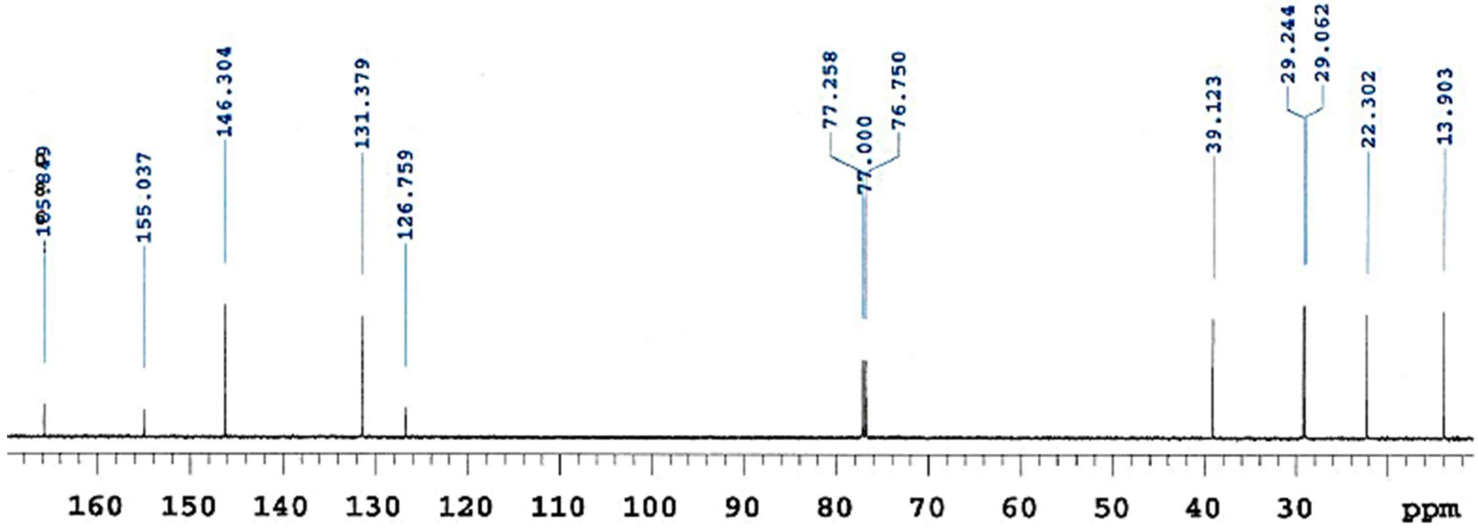

155.037
146.304
131.379
126.759
77.258
77.000
76.750
39.123
29.244
29.062
22.302
13.903
160
150
140
130
120
110
100
90
80
70
60
50
40
30
ppm

^{1}H NMR and ^{13}C NMR spectra of compound **14**

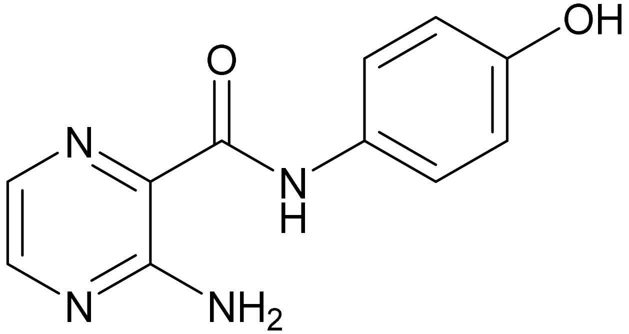

– Phenyl Group –

Compound (14)

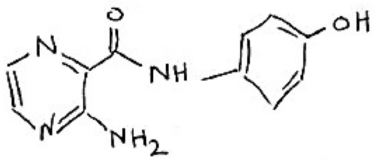

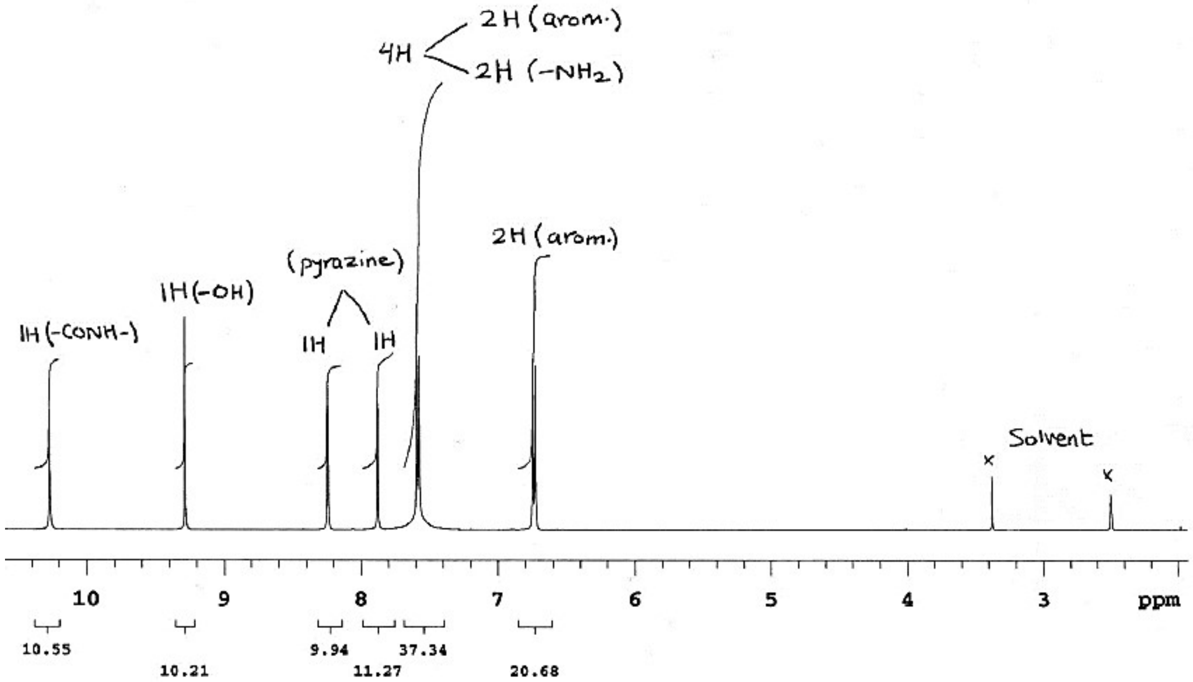

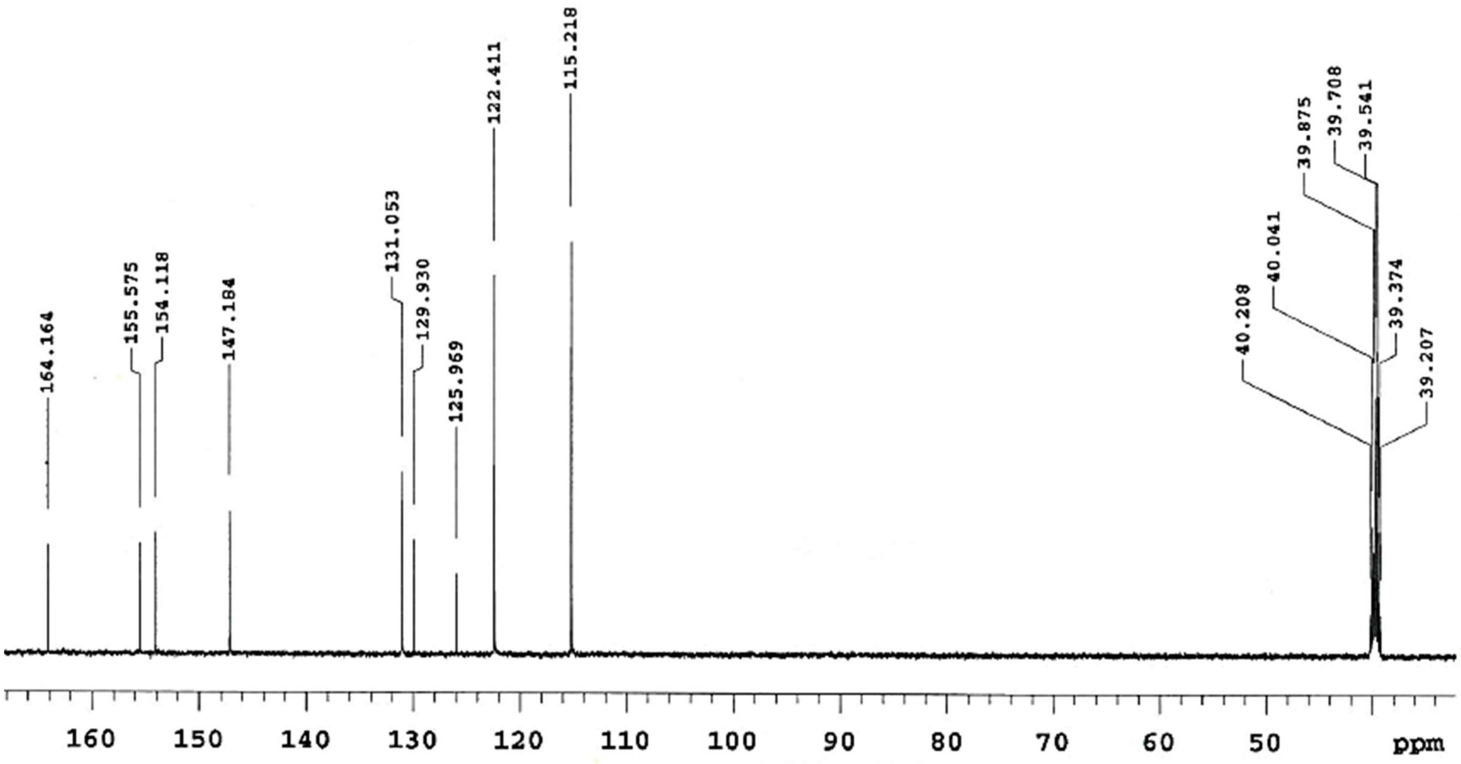
164.164
155.575
154.118
147.184
131.053
129.930
125.969
122.411
115.218
40.208
40.041
39.875
39.708
39.541
39.374
39.207
160
150
140
130
120
110
100
90
80
70
60
50
ppm
